# Analysis of cancer risk and *BRCA1 *and *BRCA2 *mutation prevalence in the kConFab familial breast cancer resource

**DOI:** 10.1186/bcr1377

**Published:** 2006-02-13

**Authors:** Graham J Mann, Heather Thorne, Rosemary L Balleine, Phyllis N Butow, Christine L Clarke, Edward Edkins, Gerda M Evans, Sián Fereday, Eric Haan, Michael Gattas, Graham G Giles, Jack Goldblatt, John L Hopper, Judy Kirk, Jennifer A Leary, Geoffrey Lindeman, Eveline Niedermayr, Kelly-Anne Phillips, Sandra Picken, Gulietta M Pupo, Christobel Saunders, Clare L Scott, Amanda B Spurdle, Graeme Suthers, Kathy Tucker, Georgia Chenevix-Trench

**Affiliations:** 1 Westmead Institute for Cancer Research, University of Sydney at Westmead Millennium Institute, Westmead Hospital, Westmead, New South Wales, Australia; 2 Research Division, Peter MacCallum Cancer Centre, East Melbourne, Victoria, Australia; 3 School of Psychology, University of Sydney, NSW, Australia; 4 Women's and Children's Health Service, Subiaco and Centre for Human Genetics, Edith Cowen University, Joondalup, Western Australia, Australia; 5 Breast Cancer Network Australia, Camberwell, Victoria, Australia; 6 Cancer Epidemiology Centre, The Cancer Council Victoria, Melbourne, Victoria, Australia; 7 SA Clinical Genetics Service, Department of Genetic Medicine, Women's and Children's Hospital, North Adelaide, Australia, and Department of Paediatrics, University of Adelaide, Australia; 8 Queensland Clinical Genetics Service, Royal Children's Hospital, Brisbane, Herston, Australia; 9 Genetic Services of Western Australia, King Edward's Memorial Hospital, School of Paediatrics and Child Health, University of Western Australia, Perth, Subiaco, Australia; 10 Centre for Genetic Epidemiology, University of Melbourne, Melbourne, Victoria, Australia; 11 Familial Cancer Centre, Royal Melbourne Hospital, Parkville, Victoria, Australia; 12 Division of Hematology and Medical Oncology, Peter MacCallum Cancer Centre, East Melbourne, Victoria, Australia; 13 School of Surgery and Pathology, University of Western Australia, Perth, Western Australia, Australia; 14 Walter and Eliza Hall Institute of Medical Research, Royal Parade, Parkville, Victoria, Australia; 15 The Queensland Institute of Medical Research, Brisbane, Queensland, Australia; 16 Hereditary Cancer Clinic, Prince of Wales Hospital, Sydney, Australia

## Abstract

**Introduction:**

The Kathleen Cuningham Foundation Consortium for Research into Familial Breast Cancer (kConFab) is a multidisciplinary, collaborative framework for the investigation of familial breast cancer. Based in Australia, the primary aim of kConFab is to facilitate high-quality research by amassing a large and comprehensive resource of epidemiological and clinical data with biospecimens from individuals at high risk of breast and/or ovarian cancer, and from their close relatives.

**Methods:**

Epidemiological, family history and lifestyle data, as well as biospecimens, are collected from multiple-case breast cancer families ascertained through family cancer clinics in Australia and New Zealand. We used the Tyrer-Cuzick algorithms to assess the prospective risk of breast cancer in women in the kConFab cohort who were unaffected with breast cancer at the time of enrolment in the study.

**Results:**

Of kConFab's first 822 families, 518 families had multiple cases of female breast cancer alone, 239 had cases of female breast and ovarian cancer, 37 had cases of female and male breast cancer, and 14 had both ovarian cancer as well as male and female breast cancer. Data are currently held for 11,422 people and germline DNAs for 7,389. Among the 812 families with at least one germline sample collected, the mean number of germline DNA samples collected per family is nine. Of the 747 families that have undergone some form of mutation screening, 229 (31%) carry a pathogenic or splice-site mutation in *BRCA1 *or *BRCA2*. Germline DNAs and data are stored from 773 proven carriers of *BRCA1 *or *BRCA1 *mutations. kConFab's fresh tissue bank includes 253 specimens of breast or ovarian tissue – both normal and malignant – including 126 from carriers of *BRCA1 *or *BRCA2 *mutations.

**Conclusion:**

These kConFab resources are available to researchers anywhere in the world, who may apply to kConFab for biospecimens and data for use in ethically approved, peer-reviewed projects. A high calculated risk from the Tyrer-Cuzick algorithms correlated closely with the subsequent occurrence of breast cancer in *BRCA1 *and *BRCA2 *mutation positive families, but this was less evident in families in which no pathogenic *BRCA1 *or *BRCA2 *mutation has been detected.

## Introduction

Ten to fifteen percent of women with breast cancer also have close relatives affected, and in many multiple-case families susceptibility to the disease appears to manifest as a dominantly inherited Mendelian trait. The *BRCA1 *locus on chromosome 17 co-segregates with breast cancer and is characteristic of families in which both early-onset breast and ovarian cancers occur [1]. A similar proportion of breast cancer families, especially those that include one or more cases of male breast cancer, is linked to a second locus (*BRCA2*) on chromosome 13 [2,3].

In the ten years since *BRCA1 *and *BRCA2 *were cloned and sequenced, thousands of pathogenic mutations have been identified. The original expectation was that carriers of these mutations would have very high (>80%) lifetime risks of breast cancer. Such dramatic effects seem to be confined to families with six or more cases of breast cancer or with a combination of breast, ovarian and male breast cancers. Population-based studies show that the average risk to carriers (40% to 65%) may be somewhat less than in multiple-case families and may vary between populations [1,4,5]. The prevalence of *BRCA1 *and *BRCA*2 mutations (approximately 1:500, but higher in Ashkenazi Jewish individuals) indicates that they are unlikely to account for more than 3% to 5% of all breast cancers. However, mutation carriers from multiple-case families constitute cohorts in which hypotheses about the role of potential modifying genes and non-genetic risk factors can be tested. Such analyses need to be complemented by population-based studies so that estimates of the prevalence, attributable fraction, and risk of breast cancer can be made for individuals who have not been selected on the basis of a strong family history [4].

Mutations in *BRCA1 *and *BRCA2 *are present in only 35% to 40% of concentrated family clusters of breast/ovarian cancer; hence it is likely that other 'high-penetrance' breast cancer susceptibility genes remain to be identified. Multiple-case families also have the potential, given sufficient numbers, to identify genes, such as *CHEK2*, that confer lower levels of risk than *BRCA1 *and *BRCA2*, and that are more prevalent in the population [6].

For these and other reasons, a series of discussions within the Australian cancer genetics and genetic epidemiology community in 1995 led to the formation of a national consortium. Its underlying goal was to foster research into the causes and impact of familial breast cancer. This would be achieved through construction of a research resource of genetic, epidemiological and clinical data, with appropriate biospecimens, to be available to researchers from anywhere in the world. From its inception the consortium developed important links with the network of family cancer clinics (FCCs) around Australia, facilitating recruitment into The Kathleen Cuningham Consortium for Research into Familial Breast Cancer (kConFab) and transfer of research findings to participants and their families. kConFab received its first funding in 1997 from the Kathleen Cuningham Foundation [7,8]. We report here on the progress to date of the consortium, and the characteristics of its first 822 multiple-case families. We demonstrate that the Tyrer-Cuzick algorithm effectively predicted breast cancer occurrence in women unaffected at the time of recruitment if they were in a family carrying a *BRCA1 *or *BRCA2 *mutation, though not otherwise. In addition, for these unaffected women, calculated probabilities of a pathogenic mutation in *BRCA1 *or *BRCA2 *were moderately predictive of such a mutation being found.

## Materials and Methods

### kConFab: structure and funding

The aims of kConFab are to collect relevant data and biospecimens from families with multiple cases of breast and/or ovarian cancer and to make these widely available, not only to its members, but to researchers from anywhere in the world for use in peer-reviewed, ethically approved research projects. To further enrich the resource, data from these research projects are returned to kConFab after publication. kConFab's policies are set by an executive committee, whose membership includes a consumer representative. Detailed policies and procedures and reports of progress are available at the kConFab website [9].

### Ascertainment of probands and family members

Since 1997, kConFab has been recruiting multiple individuals (affected and unaffected by cancer) from families that have presented to FCCs with evidence of high familial risk of breast cancer. The eligibility criteria for recruitment of families into kConFab have evolved over time but are intended to maximize the number of living potentially high-risk individuals, including carriers of high-penetrance alleles, whether affected by breast cancer or not. Families are recruited by research nurses located in all the largest FCCs in Australia and New Zealand into five categories described in Table 1.

The families are identified and initially evaluated as part of clinical care in an FCC. The de-identified pedigree is submitted to kConFab for review and, if judged eligible, the research nurse approaches a clinic-nominated Family Contact Person. Additional family members are invited to participate only with the permission of this person, or another relative subsequently recruited to kConFab. Participants can be identified from other sources, for example, by surgeons treating breast cancer patients from families who appear to fit the 'potentially high risk' category, although recruitment is again via the kConFab research nurse.

After completion of recruitment and data collection, including verification of the family history of cancer, the pedigree is again reviewed and the final category recorded. The apparent strength of family history may have altered as a consequence of verification of details, and so a proportion of families collected do not fit any category on completion (category 0), most often because insufficient eligible individuals are alive. These families are not discarded as later follow-up may provide further information that may change their category. Breast, ovarian and other cancers are regarded as 'sporadic' if they affect individuals in mutation-negative families who are unrelated by descent to other cases. Most 'sporadic' cancers occur in spouses who do not themselves appear to have a maternal or paternal history of breast or ovarian cancer. 'Sporadic' cases are excluded from the data presented in the tables. The youngest participating individual in the family affected by breast cancer is designated as the index case for the purposes of mutation testing.

### Protocols and procedures

#### Recruitment and follow-up

Each eligible subject is approached by the research nurse via the Family Contact Person or another family member and their informed consent is obtained for participation in kConFab. The research nurse invites participation by all family members reported to have been affected by breast or ovarian cancer, their first-degree relatives, all individuals in ancestral line between affected individuals and both parents of any of these eligible individuals. Each subject is then interviewed to establish their family structure and any reported diagnoses of cancer for themselves or eligible relatives. In 2003, the research nurses began annual follow-up of families via the Family Contact Person. The main aim is to offer participation to individuals who have become eligible for kConFab since the family collection was completed, including newly affected family members, children who have attained age of majority since the family ascertainment was completed and first degree relatives of participants who have recently become affected with breast or ovarian cancer. The nurses also approach first-degree relatives of men and women who have learned (through attendance at a FCC) that they carry a *BRCA1 *or *BRCA2 *mutation and notify the kConFab research nurse of this. The approach to all new participants is always mediated through an already-consented individual.

#### Epidemiological risk factor questionnaire

Questionnaires [10-13] covering a broad spectrum of risk factors are completed by telephone, and appropriate elements are collected by proxy for deceased eligible individuals.

### Biospecimens

#### Blood collection and processing

A 20 ml sample of anticoagulated blood is collected from all participants and returned to the central core laboratory by courier. Participants who are unable to give blood are mailed a mouthwash kit [14] and 73% of those from whom mouthwash samples have been requested have returned samples. The blood processing protocol [15] generates a nucleated cell product for DNA extraction, Ficoll-hypaque separated lymphocytes for Epstein Barr virus transformation, non-lymphocytic leukocytes for further DNA or RNA extraction, Guthrie cards of blood spots, and plasma aliquots. DNA is extracted as required (QIAamp DNA blood kit, Qiagen GmbH, Hilden, Germany). Lymphoblastoid cell lines are established by Epstein Barr virus transformation as required for research projects or to replace DNA stocks as they are depleted.

#### Collection of fresh normal and tumor specimens

Subjects are encouraged to report if and when they are to have breast or ovarian surgery for prophylaxis or cancer treatment. Chilled specimens are grossly dissected into 3 mm sections by the clinical pathologist, placed in a histocassette and snap frozen in liquid nitrogen and transferred on dry ice by courier to the kConFab tissue bank. In the case of prophylactic mastectomy, portions containing connective tissue are separated from fat as much as possible and marked as such by the pathologist. In remote locations, or where dry ice is not readily obtainable, specimens are collected and transported in RNA*later *(Ambion, Austin, TX, USA). A *pro forma *questionnaire about menstrual status is completed by women who donate tissues.

All material received in the bank undergoes standardized evaluation and processing, including a report on transfer conditions and state of the shipment on arrival, an assessment of the nature of the specimen and a record of number and size of pieces. After the final histopathology report is obtained, the frozen sections of the sample are examined after haematoxylin and eosin staining (5 μm sections) and five thick (10 μm) sections are then stored in a cryovial in liquid nitrogen. A further 'bookend' thin section is taken for haematoxylin and eosin staining to document the tumor content of the next level of the specimen.

#### Cancer verification

kConFab uses several methods to verify all reports of cancer in the family: through medical records or state-based cancer registries and by systematic searches by the Database Manager of Australian cancer registry records of all eligible consented living participants and deceased family members. The state-based cancer registries have been collecting data since 1982 (and some back to 1970), but most have only recorded ductal carcinoma *in situ *since 1982. The final level of verification that is reached is noted and, where possible, a copy of the final pathology report is obtained from which the location of archival, diagnostic tumor specimens is recorded so that paraffin blocks and slides can be requested as necessary.

### Genotyping

#### Mutation detection

Testing for *BRCA1 *and *BRCA2 *mutations has been performed in 747/822 (91%) of families. In most cases the enrolling FCC will already have undertaken diagnostic testing using a clinical DNA sample from an affected family member according to their local protocols, which were estimated to be about 80% sensitive prior to 2001. Since 2001 most diagnostic laboratories in Australia have adopted denaturing high performance liquid chromatography as the method of choice for *BRCA1 *and *BRCA2 *mutation analysis, with estimated 96% sensitivity for exonic and splice-junction variants [16]. Testing of index cases in kConFab families has also been carried out by complete sequencing through Myriad Genetics (Salt Lake City, UT, USA) with the support of a grant from the National Cancer Institute to the Australian Breast Cancer Family Registry [11]. Families prioritized for complete sequence analysis are those with no previous clinical mutation testing because, for example, no affected member of the family wanted a clinical mutation test result, or because the family fulfill the entry requirements of a genome-wide linkage study being undertaken in collaboration with the Breast Cancer Linkage Consortium [1]. In addition, all individuals who have provided a fresh frozen breast tumor (cancer) specimen are tested by full sequencing unless they are already known to be positive for a family-specific mutation in *BRCA1 *or *BRCA2*. Analysis for genomic deletions and rearrangements detectable by multiplex ligation-dependent probe amplification [17] is currently underway.

kConFab classifies all *BRCA1 *and *BRCA2 *variants reported by diagnostic or research laboratories into categories: pathogenic, splice-site variant, variant of unknown significance and polymorphism. The criteria for classification are posted on kConFab's website [18].

Once the family mutation has been identified, all pathogenic (including splice site) variants of *BRCA1 *and *BRCA2 *are genotyped by kConFab in all available family members' DNA. Access to the unique family number linking a variant to a particular family is password protected and is only available to investigators carrying out kConFab-related research. De-identified, individual mutation results are available only to researchers, and not to the originating research nurse(s) or health professionals in the FCC(s) that the family attends.

All index cases of non-*BRCA1/2 *families are genotyped by kConFab for the breast-cancer related mutation in *ATM*, 7271T>G [19], and a small number of families with features of Li Fraumeni syndrome have been tested for mutations in p53 by the diagnostic laboratories.

#### Haplotyping at *BRCA1 *and *BRCA2 *loci

Mutation-negative families with female breast cancer, in which sufficient quantities of DNA are available from at least three affected individuals, have been analyzed for haplotype sharing at short tandem repeat polymorphism (STRP) markers flanking and internal to *BRCA*1 (D17S800, D17S855, D17S951, D17S1322) and *BRCA*2 (D13S260, D13S1700, D13S171, D13S267) in order to select families suitable for genetic linkage analysis for novel susceptibility loci.

### Informatics

The Central Register, located at the Peter MacCallum Cancer Centre, until recently received data from peripheral versions of a Family Based Information database, developed by the Cancer Council of Victoria, by periodic uploads from the recruiting clinic-based research nurses. During 2005, we implemented a TCP-IP based application to permit live access to the central data server from multiple remote locations, based on a Progeny Anywhere (Progeny Software LLC, Grand Rapids, MI, USA) XML-compliant front end. Data in the Central Register can be accessed only by procedures that are ethically approved and in accord with national guidelines on privacy of genetic information [20]. Information is stored both by individual and by family. Individual data include personal and demographic information, biological relationships, cancer diagnoses, cancer treatments, epidemiological information, biological specimens, genotyping and mutation test results, as well as a variety of process and management outcomes.

Periodic checks are run to determine if individuals have been registered through more than one family and, if confirmed and appropriate to the family structure, the families are merged within the Central Register. In this way, and through other consent-approved communication between family cancer clinics, 43 families with individuals in common have been merged into 21, ensuring data and specimens for each individual are only accessed once, and that the combined characteristics of the entire family structure can be analyzed by researchers.

### Quality control

Identity and DNA quality are checked on a randomly selected 10% of the DNAs extracted from leukocyte pellets by PCR amplification of five unlinked STRP markers. These genotypes are compared with those from DNA from the matching Guthrie spot. To date, all DNAs tested in this way have yielded PCR product, and none have shown mismatches with the Guthrie spot DNA.

After its initial submission, and whenever genotyping has been extended within a family as a result of a newly detected mutation, the pedigree is reviewed by a geneticist for internal consistency of the family structure, dates of birth, cancer diagnosis and death, and for Mendelian inheritance of known genotypes. Using DNA isolated from a Guthrie spot, we repeat testing for the family-specific mutation in any individuals with apparently anomalous results; for example, unaffected but mutation-positive individuals with no confirmed carrier or affected offspring, who were linked to the closest confirmed carrier by two or more unaffected individuals of unknown genotype. To date, these and other types of quality control have not revealed any genotyping errors.

### Pathology review

A standardized review of original or duplicate diagnostic slides from all index cases in families in which no mutation in *BRCA1*/2 had been identified was begun in 2004 with the aim of identifying those cases with a phenotype suggestive of a *BRCA1 *mutation in order to direct additional mutation testing, and to determine if there are distinct pathological phenotypes by which these families could be stratified. The review, aided by a reference document, involves recording tumor size, lymph node and hormone receptor status from original pathology reports and examining slides to evaluate tumor type and grade as well as features of particular significance to familial breast cancer such as lymphocytic infiltrate and the extent of a pushing tumor margin [21].

### Communication with subjects

kConFab maintains contact with participants, collaborating doctors, pathology laboratories and funding bodies through a newsletter, published once or twice yearly, describing kConFab's progress and providing other general information about familial breast and ovarian cancer. In addition, as the results of genetic tests performed by kConFab become available, participants are contacted if they have indicated a wish to be informed 'if there is a test result (obtained by kConFab) that may have implications for me or my family'. This process is activated when a mutation of pathogenic significance has been identified in a member of the family, and all available samples from the family have been genotyped. kConFab's letter to participants does not provide an individual result, but rather says that genetic information 'relevant to the family' is now available. The letter goes on to explain how and where to seek further advice and clinical testing in an accredited laboratory via a FCC. kConFab notifies the relevant diagnostic laboratory of the nature of the family mutation, but does not supply individual research results to the laboratory.

### Ethics

kConFab maintains human ethics approval at all participating institutions through which subjects are recruited. All research projects making use of data and/or materials collected by kConFab are required to have independent ethical approval from their host institutions. All participants give informed consent and understand that as a result of participation, personal details will be recorded and stored in a coded format on a database. They consent to samples of genetic material, blood cells and tissue (if applicable), being stored in a central location and to de-identified information and samples being made available for scientifically and ethically approved research projects.

### Access to data and biospecimens

Applicants wishing to use data and/or biospecimens first submit a brief expression of interest, which is circulated to the entire kConFab membership with a five-day opportunity given to highlight major issues, especially duplication of, or complementarity to, existing projects. A full application is then submitted and checked against criteria that include evidence of external peer review, sufficient resources to conduct the project, and ethics approval. Given that these conditions are met, approval is given for up to three years, during which annual progress reports are a requirement, and after which the project can be renewed if not complete. To further enrich the kConFab resource, the researchers are required to supply their research data to kConFab after publication, and/or 12 months after completion of their project [22].

### Analysis of breast cancer risk

The Tyrer-Cuzick algorithm [23] was developed to model breast cancer risk in unaffected women by taking into account: their probability of carrying genetic risk factors, namely a rare, high penetrance mutation in *BRCA1 *or *BRCA2 *and a notional common, low-penetrance dominant susceptibility allele that stood for all other genetic risk factors; and a range of other individual clinical and epidemiological factors known to influence risk, such as menarche and parity. The genetic risk component of the calculations derives from a Bayesian segregation analysis that takes into account known *BRCA1/2 *genotyping data and family history of breast cancer in first- and second-degree relatives.

A batch program kindly provided by Dr Jack Cuzick [23] was used to estimate the risk of breast cancer in the next decade for all women in the kConFab cohort who had not previously had breast cancer at time of recruitment. The reference date for the subject was the date of epidemiological risk factor interview, but the family data used (family structure and cancer diagnoses in relatives) were those that had been reported in the family by the date of analysis, not only those reported at the time of interview. Two calculations were performed, one in which all reports of previous family breast cancer diagnoses were taken into account, and one including only the verified reports. The program was also used to calculate the probability of testing positive for a *BRCA1 *or *BRCA2 *mutation. It is recognized that the circumstances of genetic testing of individuals in the kConFab cohort were often far from those for which this aspect of the algorithm was designed, namely the initial screening of an index case in a previously untested family. Receiver operating characteristic (ROC) analysis of the sensitivity and specificity of these estimates was performed using STATA 7.0 (College Station, TX, USA).

## Results and Discussion

### Family characteristics and biospecimen collection

The majority (518; 63%) of the 822 families in this cohort have cases of female breast cancer in successive generations, but no cases of ovarian or male breast cancer (Table 2). Families having both female breast and ovarian cancer account for most of the remaining families (239; 29%). Male breast cancers were reported in only 54 families (7%). The median number of breast or ovarian cancers per family was 4 (range 0 to 19), with 691 (84%) families having at least three and 191 (23%) having at least five.

Pathogenic (including splice site) mutations in *BRCA1 *or *BRCA2 *have been detected in 229 families. There are 62 families with only unclassified variants in *BRCA1 *or *BRCA2*, while six families have variants that have not yet been categorized. In the 525 families without a *BRCA1 *or *BRCA2 *pathogenic, splice-site mutation or unclassified variant, genetic testing has been completed on at least one member of the family for 136 families, with 314 having had partial testing, and 75 no testing. The median age at diagnosis of female breast cancer, male breast cancer and ovarian cancer in *BRCA1 *and *BRCA2 *carriers or pathogenic or unclassified variants and those without identified variants are given in Table 3. As expected, the median age at diagnosis of the first female breast cancer was lower in *BRCA1 *and *BRCA2 *carriers than in those women without mutations.

Although testing is incomplete, only two families (Table 1, category 3) have identifiable mutations in genes other than *BRCA1 *or *BRCA2 *that are likely to have conferred a high risk of breast cancer. One family has a mutation in *TP53* and another has a 7271T>G mutation in *ATM *[19] (Table 4). Twenty-five potentially high-risk families were enrolled into kConFab (Table 1) because a family member wanted to give fresh surgical tissue, but the family did not fit other kConFab ascertainment criteria.

Germline DNA samples have been collected from 7,389 individuals (Table 4). Epidemiological questionnaires have been collected from 7,039 individuals, with more limited data concerning a further 4,383 deceased relatives collected by proxy (Table 4). Participation has been greater by female than male family members, with females accounting for 65% of the biospecimens and 64% of the questionnaires provided.

The mean number of germline DNA samples collected per family is nine (median of eight; Table 5). From 122 families, germline DNA samples were collected from three or more affected individuals (Table 5). Lymphoblastoid cell lines are currently available for 462 subjects, and can be made from any consented individuals from whom at least 10 ml blood was collected (92% of all blood samples).

Verification was obtained for 1,808/3,135 (57.7%) reported female breast cancers, 159/329 (48.3%) ovarian cancers, 27/60 (45.0%) male breast cancers and 690/2,580 (26.7%) other cancers. Family review identified 37 'sporadic' cases in mutation-negative families, from 7 of whom we have germline DNA. Fresh tissue has been frozen (or stored in RNA*Later *for 11%) for 253 specimens (Table 6). These include 45 breast tumors, 6 ovarian tumors, and 75 normal breast and 73 normal ovary specimens from prophylactic surgery.

### *BRCA1 *and *BRCA2 *mutations

*BRCA1 *and *BRCA2 *variants identified in kConFab families are listed on the website [18]. Pathogenic (including splice-site) *BRCA1 *mutations were detected in 122 of the families and variants of uncertain significance (unclassified variants) in 26 (Table 2). Pathogenic (including splice-site) *BRCA*2 mutations were detected in 107 families and unclassified variants in 36. The frequency of pathogenic and splice-site mutations in *BRCA1 *or *BRCA*2 in families that had had at least some mutation screening was 31% (229/747), while unclassified variants were found in a further 8% (62/747). Even after mutation screening of selected families by full sequencing, only 51% (229/453) families were found to carry *BRCA1 *or *BRCA2 *mutations. However, this figure is likely to increase as testing (including screening for large genomic rearrangements) is completed across the entire cohort. Biospecimens and data are available from 773 proven carriers of *BRCA1 *or *BRCA2 *pathogenic and splice site mutations (including 558 females, of whom 342 are affected by breast or ovarian cancer), and data from a further 127 obligate carriers (93 female, including 81 affected) (Table 7). There were 15 cases from mutation-positive families with female breast or ovarian cancer who did not carry the family mutation. *BRCA1 *and *BRCA2 *mutation status is known for approximately half the donors of fresh tissue biospecimens (Table 6). For example, there are 16 breast tumors, 34 prophylactic mastectomy specimens and 53 prophylactic oophorectomy specimens from proven *BRCA1 *and *BRCA2 *carriers in the tissue bank.

Because the initial screening for *BRCA1 *and *BRCA2 *mutations was performed by local diagnostic laboratories using protocols of varying sensitivity, a systematic attempt is underway to identify additional mutations using full sequencing of *BRCA1 *and *BRCA2*, which has been performed in 168 cases (from 163 families). Therefore, 178 families are regarded as 'non-*BRCA1/2*' by sensitive testing methods, although 42/178 have unclassified variants in *BRCA1 *or *BRCA2*.

To date, 20 families carry mutations that are believed to have resulted from *Alu *recombination events resulting in large genomic deletions and rearrangements in *BRCA1*. The common and well-characterized mutation *BRCA1 *exon 13 duplication accounts for 8 of these families and 12 families carry variants of either single or multiple exon deletions and duplications. The five families whose genomic rearrangements have not yet been fully characterized are not included as mutation-positive in the tables.

Genotyping has been used to select families suitable for genetic linkage analysis for non-*BRCA1*, non-*BRCA2 *high penetrance susceptibility loci, namely those in which all affected individuals do not share a haplotype at *BRCA1 *or *BRCA2 *(Table 8). Haplotyping has also been useful in redirecting mutation analysis, for example if the majority of affected individuals in the family shared a *BRCA1 *or *BRCA2 *haplotype but the screened individual(s) did not, and to prioritize families for more intensive mutation analysis such as full sequencing or genomic rearrangement analysis. Where germline DNA samples were available from at least three affected individuals, haplotyping at STRP markers flanking *BRCA1 *and *BRCA2 *was carried out in *BRCA1*, *BRCA2 *mutation-negative families.

### Research projects supported

Since 1998, 45/48 research project applications have been given approval to use kConFab biospecimens and data, one third of these in the last two years. The average time from receipt of an application to approval of the project has been four months, which usually includes some clarification of the protocol with the applicant. Three projects did not go ahead because of lack of funding or the unwillingness of the host institution to sign a materials transfer agreement. Active research projects are listed on the kConFab website [24]. The investigators involved come from multiple institutions in Australia, one in the United States, one in the United Kingdom and one in France. To date, these projects have resulted in 29 primary research publications [25], ranging from reports on the cumulative risk of breast cancer in families with *BRCA1 *and *BRCA2 *mutations to studies of psychological morbidity in these families [8,19,26-37].

### Quantification of breast cancer risk

The risk of breast cancer in the ten years after recruitment was calculated for all 4,815 unaffected female kConFab subjects in which epidemiological interview data, collected directly or by proxy, were available. This risk was expressed as either the absolute risk or as a ratio of population risk for that individual (relative risk).

In the cohort as a whole, the distribution of relative risk indicated that our ascertainment scheme and procedures had been reasonably efficient. The median relative risk was 2.47, with 91% of subjects having a risk higher than that of a woman in the population of the same age and 859 (18%) having a more than 5-fold elevation.

**Figure 1:**
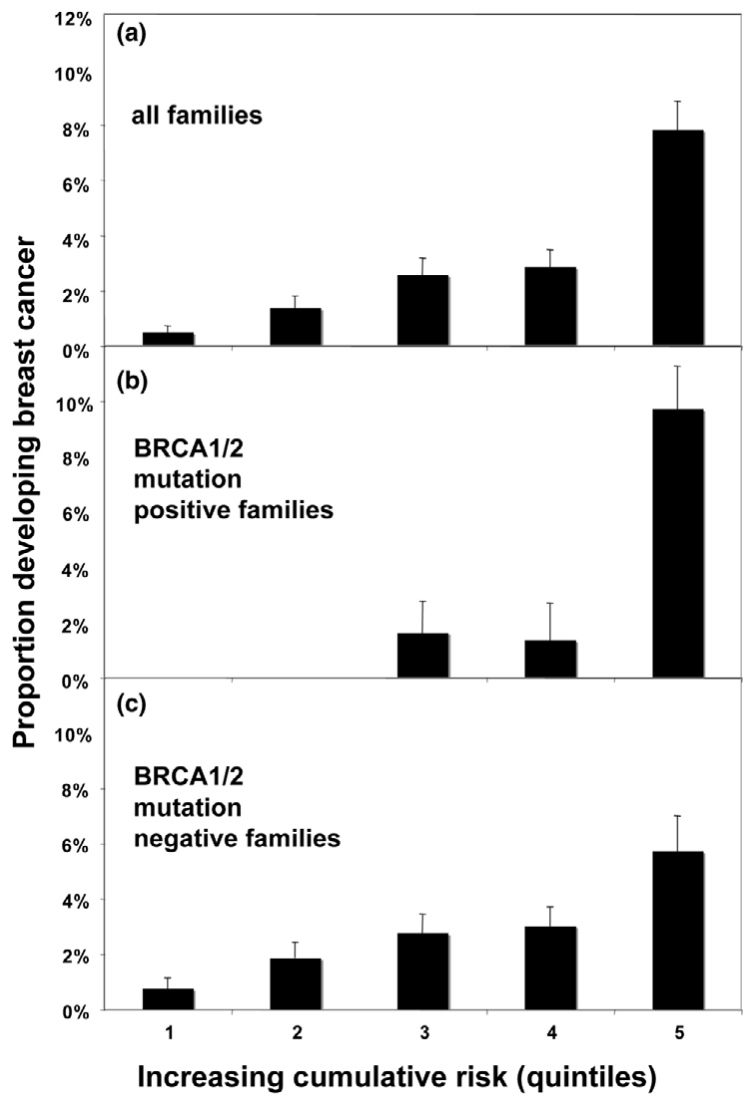
Relationship between estimated cumulative probability and actual relative frequency of breast cancer since ascertainment. Women unaffected at time of interview were ranked in quintiles according to their cumulative risk of breast cancer since interview, calculated from the absolute risk estimated by a batch program implementing the Tyrer-Cuzick algorithm. The proportion of women in each risk quintile (horizontal axis: 1 lowest to 5 highest) developing breast cancer during a median 4.2 years follow-up is plotted on the vertical axis plus standard error. **(a) **All families; **(b) ***BRCA1 *or *BRCA2 *mutation positive families; **(c) ***BRCA1 *or *BRCA2 *mutation negative families.

The graphs in Fig. 1 show the relationship between the probability of having developed breast cancer for the first time since interview and the cumulative risk over the period of follow-up as calculated using the Tyrer-Cuzick model. Over this time (median 4.2 years) there were 107 cases of breast cancer confirmed by medical records, 2.9% of the 3,657 women alive at the time of initial data collection. This was very close to the 110 cases expected. Women in the highest risk quintile (absolute risk > 0.14, relative risk > 10.1) accounted for 49 of those cancers (46%, data not shown). Overall, 55 cancers (51%) occurred in women in the highest quintile of cumulative risk, in which length of follow-up was taken into account (Fig. 1a). In families with a pathogenic *BRCA1 *or *BRCA2 *mutation, women in the highest quintile of cumulative risk experienced almost all, 92% (36/39), of the cancers (Fig. 1b). In contrast, families without a *BRCA1 *or *BRCA2 *mutation showed a weaker, but more linear relationship between calculated cumulative risk and the probability of subsequent breast cancer (Fig. 1c). Interestingly, there was no gain in predictive power when the cancer diagnoses in family members, used in the risk calculation, were restricted to those verified from medical records rather than using all reports (data not shown). For example, women in the highest quintile of cumulative risk experienced 55 of the 107 breast cancers when the verified data were used, exactly as above. The data for *BRCA1 *and *BRCA2 *mutation positive families, using verified data only, were also essentially identical to those in Fig. 1b.

High risks calculated by the Tyrer-Cuzick algorithm therefore correlated well with future incidence of breast cancer, but this effect was much stronger in *BRCA1*/2 mutation positive families than in *BRCA1*/2 mutation negative families.

### Prediction of *BRCA1 *and *BRCA2 *mutation status

**Figure 2:**
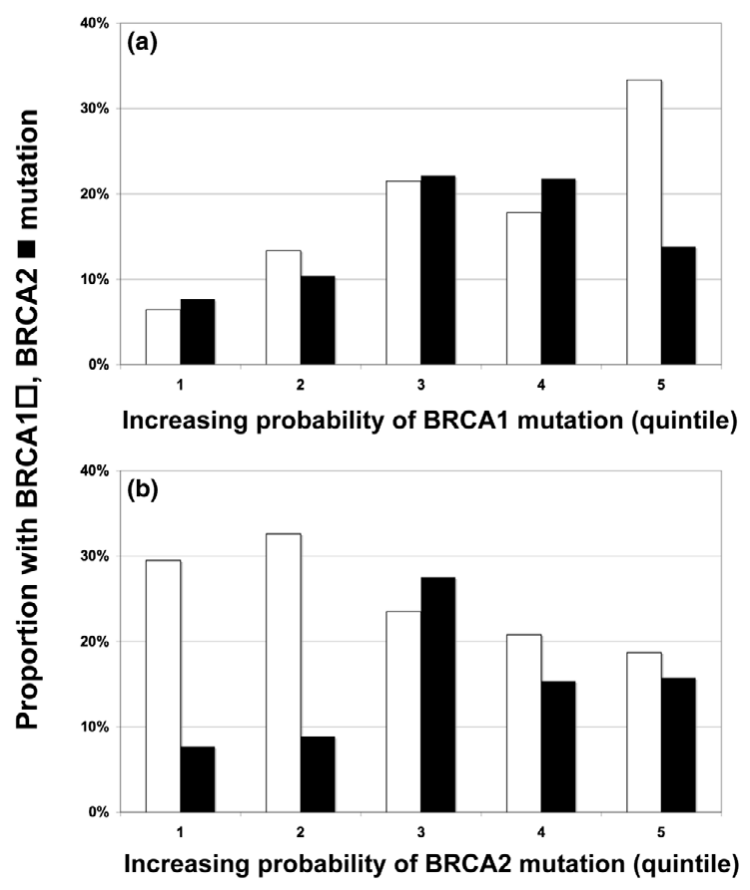
Relationship between estimated probability and predicted relative frequency or BRCA1 and BRCA2 mutations. Women unaffected at time of interview were ranked in quintiles according to their probability of carrying either a **(a) ***BRCA1 *or **(b) ***BRCA2 *mutation, as estimated by a batch program implementing the Tyrer-Cuzick algorithm, without input of family or individual mutation status. The proportion of women that tested positive for either a *BRCA1 *(open bars) or *BRCA2 *(closed bars) mutation is plotted on the vertical axis.

The Tyrer-Cuzick batch program was also used to calculate the posterior probabilities that an unaffected woman was a carrier of a *BRCA1 *or *BRCA2 *mutation. These estimates were compared with the actual prevalence of *BRCA1 *and *BRCA2 *mutations in these individuals. No data on the presence of these mutations were used in the generation of the estimates. This aspect of the algorithm was developed for use in the context of an unaffected woman presenting from a family in which no mutation screening had been done. Its application to all unaffected women in the kConFab cohort is somewhat artificial because in many cases they were recruited into kConFab after a family mutation had already been found. Nonetheless, the proportion of women testing positive for a *BRCA1 *mutation is seen to correlate well with the estimated probability of *BRCA1 *mutation (Fig. 2a). The quintile with the highest probability showed more than six times the prevalence of *BRCA1 *mutations in the lowest quintile; the area under the ROC curve was 0.67. There was also positive correlation between the *BRCA1 *estimate and the observed prevalence of *BRCA2 *mutations (Fig. 2a). Interestingly, the estimated probability of *BRCA2 *mutation was somewhat less effective than the estimated probability of *BRCA1 *mutation in predicting the presence of a *BRCA2 *mutation (Fig. 2b): the area under the ROC curve was 0.61.

## Conclusion

Knowledge of the underlying determinants of breast and ovarian cancer risk is still very incomplete. kConFab has therefore focused on recruitment and comprehensive characterization of a cohort of women at significantly higher than average risk of breast and/or ovarian cancer, together with large numbers of their relatives. This approach has already been productive in yielding many carriers of known predisposing mutations, and it is expected that these numbers will increase as *BRCA1 *and *BRCA2 *testing is completed, and as new predisposing genes are discovered. The cohort of kConFab families is a fertile resource for research and already supports a wide range of projects. Data collection is complete for 518 families with female breast cancer only, 239 with female breast and ovarian cancer, and 54 with male breast cancer alone, and/or with female breast and ovarian cancer. Data are available for 11,422 consented participants, including 900 carriers of pathogenic or splice site *BRCA1 *or *BRCA2 *mutations, and germline DNA biospecimens for 7,389, including 773 carriers. Fresh frozen tumor and normal specimens are available for 253 participants.

Ascertainment to kConFab is not population based, but is dependent on clinical identification of individuals at high risk of breast and ovarian cancer. Data and analyses derived from cohorts of this type are shaped by the selection criteria used to refer families to FCCs, the specific kConFab ascertainment criteria, and by the dynamics of family participation in research. Such clinic-based studies cannot replace population-based approaches but are complementary to them, and this project has already provided several examples of productive collaboration [19]. However, we are only recruiting a small proportion of high-risk breast cancer families in Australia and New Zealand, and recruitment is not yet reaching a plateau. The number (1,159) of *BRCA1/2 *mutation carriers for whom data is currently held is approximately 5% of the number predicted from the plausible population frequency of these alleles and the current population size of 22 million. By the time recruitment of the kConFab cohort is complete, we estimate that we will have enrolled approximately 10% of multiple case families from Australia and New Zealand. Further analyses will be needed to test how representative the cohort is of the diverse populations of our two countries.

The establishment of an organization such as kConFab cannot be achieved without some difficulties. For example, large scale centralized pathology review of breast and other cancers occurring in kConFab participants is beyond the scope of current core activities. As a consequence, systematic review has focused on index cases of non-*BRCA1/2 *families since these data are likely to be of value to research projects. Additional pathology data are contributed to kConFab by investigators who undertake review in the context of specific research projects.

Follow-up contact with the families has also proved challenging. For example, recontacting subjects for projects that include the study of known mutation carriers requires very careful attention to ensure that the presence or absence of a family mutation is not revealed to individuals who do not know, or want to know, their mutation status. Projects that involve approaching both mutation-positive and mutation-negative members of families, and can be done blind to the mutation status, are easiest to accommodate within kConFab.

One of the most difficult issues has been to ensure that the participants understand the differences between 'research' and 'clinical' mutation testing. Anecdotal evidence suggests that many participants still confuse the issues, and expect kConFab to give them individual mutation results. Most of the people enrolled in kConFab are not clients of FCCs, but relatives of people who are. Consequently, kConFab's notification letters do not tell individuals about their own carrier status, but state that a family specific mutation has been discovered and that if the individual wishes to know his/her carrier status, he/she should contact an FCC. A second blood sample can then be taken and tested in an accredited laboratory for the presence or absence of the family specific mutation. The result of this test is then given to the individual, after counseling.

Even though kConFab's ascertainment strategy is targeted to individuals with a potentially high risk of breast or ovarian cancer and their relatives, the family pedigrees contain many family members who are not necessarily at high risk, for example, spouses with no blood relationship to the cases of breast or ovarian cancer in the pedigree. Studies based on the secondary recruitment of potentially high-risk individuals within the kConFab cohort, for example, examining the determinants of clinical outcome or of psychological adjustment to high risk of cancer, have had to take additional steps to remove such individuals from their ascertainment, including implementing a system of risk stratification within the cohort.

kConFab can be compared to the European-based Breast Cancer Linkage Consortium (BCLC), which focuses on families ascertained through family cancer clinics, and the NCI-funded Breast Cancer Family Registry (BCFR) that includes both clinic-based and population-based sites. There are enough similarities between these cohorts to allow collaborative projects to use data from each cohort. A major strength of kConFab over the BCFR is the large number of available blood samples per family, which allows for more accurate penetrance estimates, and a larger number of available samples from known mutation carriers. The BCLC families are large, but epidemiological data have not been systematically collected, nor is there a central repository of biological specimens for which researchers can apply.

The Tyrer-Cuzick algorithm was applied to the cohort to test its ability to predict future breast cancer and positive *BRCA1 *or *BRCA2 *mutation tests, with considerable success. In *BRCA1 *or *BRCA2 *mutation positive families, the highest risk quintile included almost all the women that developed breast cancer over the comparatively short period of four years of follow-up. It should be noted that in these families there was already considerable information on the presence of these mutations throughout the families, and that this clearly contributed to the high predictive power of the risk estimates. When there was no *BRCA1*/2 mutation known in the family, the algorithm stratified the women much less strongly. This could be interpreted to mean that in these mutation negative families individual non-genetic risk factors such as parity and menarche still contribute to risk in the context of a strong family history but rather more weakly, even in aggregate, than as yet undetected major genetic effects.

The future data and specimen accrual goals of kConFab include recruitment of an additional 400 families to reach our target of at least 1,200 multiple-case families, collection of annual serum and plasma samples from a subset of women at risk, more intensive mutation analysis of non-*BRCA1/2 *families, further pathology review, and the generation of tissue microarrays from tumors with known and unknown mutations. These will support increasingly intensive efforts by the international breast cancer research community to solve fundamental questions about the causation of this disease.

## Abbreviations

BCFR = Breast Cancer Family Registry; BCLC = Breast Cancer Linkage Consortium; FCC = family cancer clinic; kConFab = The Kathleen Cuningham Consortium for Research into Familial Breast Cancer; ROC = receiver operating characteristic; STRP = short tandem repeat polymorphism

## Competing interests

The authors declare that they have no competing interests.

## Authors' contributions

GJM, EN: Performed the analysis of the Tyrer-Cuzick algorithm, participated in the design and coordination, and helped to draft the manuscript. HT, RLP, PNB, CLC, EE, SF, EH, MG, GGG, JG, JLH, JK, GL, KAP, SP, CS, CSL, ABS, GS, KT, GCT: Participated in the design and coordination, and helped to draft the manuscript. GME: Community representative and participated in the design and coordination and helped to draft the manuscript. JAL: Supervised the BRCA1/2 mutation detection, participated in the design and coordination, and helped to draft the manuscript. GMP: Supervised the haplotyping of kConFab families, participated in the design and coordination and helped to draft the manuscript. All authors read and approved the final manuscript.

## Figures and Tables

**Table 1:** Ascertainment criteria for defined kConFab categories

Category	Criteria^a^
1	Families in which no predisposing mutation has been identified
	Four or more cases of breast or ovarian cancer (on one side of the family) and
	Two or more living affecteds with breast or ovarian cancer and
	Four or more living first or second degree unaffected female relatives of affected cases
	
1B	Families in which no predisposing mutation has been identified
	Two or three cases of breast or ovarian cancer (on one side of the family), if at least one of these cases is 'high risk' (i.e. male breast cancer, bilateral breast cancer, breast plus ovarian cancer in the same individual, or breast cancer with onset less than 40 years) and
	Two or more living affected cases with breast or ovarian cancer and
	Four or more living first or second degree unaffected female relatives of affected cases
	
2	Families in which a *BRCA1 *or *BRCA2 *variant has been identified
	Two or more carriers, or likely carriers of a pathogenic, splice site or unclassified variant^b^, for example, two living affecteds, or one living affected and at least two living first degree unaffected females of a proven or likely mutation carrier
	
3	Families with mutations in other breast cancer predisposing genes
	Families that carry pathogenic mutations in PTEN, TP53 or ATM, and have two or more living carriers, or potential carriers
	
4	Potentially high-risk individuals from whom fresh tumor is available, but who do not fit other criteria
	Individuals that fit the Australian National Breast Cancer Centre guidelines for high risk of breast cancer (category III) [39], but who do not fit other kConFab categories, if they wish to donate fresh breast or ovarian surgical tissue to kConFab
	
5	Families with two cases of ovarian cancer that do not fit any of the other criteria
	Two or three cases of ovarian cancer among first degree relatives, one or more of whom is alive and
	Zero or one case of breast cancer and
	Four or more living first or second degree unaffected female relatives of affected cases

^a^All criteria refer exclusively to individuals over the age of 18. ^b^Families with unclassified variants in *BRCA*1 and *BRCA*2 were included in category 2 in order to maximize the opportunities to collect material that might be used in research studies aimed at trying to classify these variants.

**Table 2:** Family characteristics: cancer type, density and *BRCA*1/2 mutation status

	Female breast cancer only	Female breast and ovarian cancer	Ovarian cancer only	Male breast cancer only	Female and male breast cancer	Female breast, male breast and ovarian cancer	No breast or ovarian cancer^a^	Total number of UFNs^b^
Total number of UFNs	518	239	4	3	37	14	7	822^c^
	Number of affecteds per family							
<3	100	14	3	3	4	0	7	131
3–5	330	143	1	0	21	5	0	500
>5	88	82	0	0	12	9	0	191
Median age at diagnosis (range)	49 (20–91)	48 (22–89)	51 (36–57)	63 (47–68)	54 (30–87)	53 (31–86)	-	49 (20–91)
Median number of individuals with breast or ovarian cancer (range)	4 (1–14)	5 (1–19)	1 (1–3)	2 (1–2)	4 (2–12)	6.5 (3–17)	-	4 (0–19)
	Number of families by *BRCA1/BRCA*2 mutation status^d^							
*BRCA1 *pathogenic or splice site mutation	42	71	2	0	1	2	4	122
*BRCA1 *unclassified variant only	19	7	0	0	0	0	0	26
*BRCA2 *pathogenic or splice site mutation	60	32	1	1	7	6	0	107
*BRCA2 *unclassified variant only	21	12	0	0	3	0	0	36
*BRCA1 *or *BRCA2 *variant not yet classified	5	5	0	0	0	0	0	6
No *BRCA1 *or *BRCA2 *mutation: complete testing^e^	84	36	0	0	12	4	0	136
No *BRCA1 *or *BRCA*2 mutation: incomplete testing	231	69	1	1	10	2	0	314
Not yet tested for *BRCA1 *or *BRCA2*	56	11	0	1	4	0	3	75

The table shows the number of tumors reported and verified in all available generations on the genetically informative side of families. ^a^Seven families had no cases of breast or ovarian cancer at the time the database was surveyed. Since then cancer diagnoses have been downloaded to the Central Registry for four families that had been downloaded prematurely, one family has been merged with another that contains cases of cancer, and two more families are category 4, with a member who wished to give fresh tissue to kConFab, but no additional information is available on their affected relatives. ^b^UFN, unique family number. ^c^Merged families are counted by their individuals components, not as a merged pedigree. ^d^Some families have a pathogenic or splice site mutation as well as an unclassified variant, and some families have two unclassified variants. ^e^At least one person tested by high-sensitivity methods for *BRCA1 *and *BRCA2 *mutations.

**Table 3:** Median age of onset of first diagnosis of breast or ovarian cancer in *BRCA1/2 *carriers and non-carriers

	*BRCA1 *carrier of a pathogenic or splice site mutation	*BRCA2 *carrier of a pathogenic or splice site mutation	*BRCA1 *carrier of an unclassified variant	*BRCA2 *carrier of an unclassified variant	No identified mutations
Females with breast cancer (range)	40 (23–70)	42 (24–79)	44 (22–88)	43 (29–79)	50 (15–90)
Females with ovarian cancer (range)	50 (33–80)	56 (45–77)	none	80 (80-80)	55.5 (27–79)
Females with breast and ovarian cancer (range)	50 (26–85)	60 (41–73)	62.5 (55–75)	none	53 (30–75)
Males with breast cancer (range)	none	68.5 (46–86)	none	none	62 (31–75)

**Table 4:** Availability of germline DNA specimens and epidemiological data

	Family ascertainment category^a^							Review pending	Total
	1	1B	2	3	4	5	0		

Number of families	292	113	217	2	25	8	140	25	822
Number of samples									
From females (affected)	2,118 (735)	577 (215)	1,239 (385)	19 (6)	45 (21)	22 (12)	621 (179)	124 (44)	4,765 (1597)
From males (affected)	1,179 (7)	274 (6)	759 (5)	5 (0)	28 (0)	14 (0)	322 (4)	43 (0)	2,624 (22)
Total^b^	3,297	851	1,998	24	73	36	943	167	7,389
Number of epidemiological questionnaires									
Females (affected)	2,033 (703)	526 (193)	1,226 (386)	18 (13)	31 (13)	18 (9)	585 (163)	100 (37)	4,537 (1510)
Males (affected)	1,122 (5)	262 (6)	745 (5)	5 (0)	18 (0)	11 (0)	303 (3)	36 (0)	2,502 (19)
Total^c^	3,155	788	1971	23	49	29	888	136	7,039^d^
Number of proxy epidemiological questionnaires									
Female (affected)	889(460)	226 (81)	694 (410)	48 (1)	25 (13)	7 (4)	382 (160)	53 (31)	2,284 (1160)
Male (affected)	862 (7)	206 (3)	608 (9)	11 (0)	18 (0)	5 (0)	336 (1)	53 (1)	2,099 (21)
Total^e^	1,751	432	1,302	19	43	12	718	106	4,383

^a^Final category (Table 1) after review. ^b^Germline DNA mainly from blood samples but includes 22 obtained from mouthwash specimens. ^c^Epidemiological questionnaires. ^d^The lower number of epidemiological questionnaires than biospecimens reflects a time-lag in collecting them from the research nurses, and scanning them. ^e^Epidemiological questionnaire data obtained for deceased individuals by proxy.

**Table 5:** Availability of germline DNA specimens by cancer family type

Type of cancer family	Mean number of blood samples per family (range)	Samples from affecteds/family (number of families)				
		0	<2	2	>2	All families

Female breast cancer only	8.6 (1–36)	98	186	166	68	518
Female breast and ovarian cancer	10.3 (1–37)	42	79	78	40	239
Ovarian cancer only	4.8 (2–7)	2	1	1	0	4
Male breast cancer only	4.3 (2–8)	0	3	0	0	3
Female and male breast cancer	8.1 (2–18)	9	12	8	8	37
Female breast, male breast and ovarian cancer	13.8 (2–30)	1	4	3	6	14
All families with breast or ovarian cancer	9.1 (1–37)	152	285	256	122	815

**Table 6:** Mutation status of individuals from whom fresh frozen normal and malignant ovarian and breast specimens have been obtained

Type of surgery/tissue	*BRCA1 *mutation positive	*BRCA2 *mutation positive	*BRCA1/2 *mutation negative^a^	Unknown	Total
Breast					
Prophylactic/tumor	0	0	1	0	1
Prophylactic/normal	18	16	2	39	75
Cancer/tumor	9	7	15	13	44
Cancer/normal	6	5	11	17	39
Ovary					
Prophylactic/tumor	0	0	0	1	1
Prophylactic/normal	27	26	0	20	73
Cancer/tumor	4	0	0	1	5
Cancer/normal	6	2	0	7	15

^a^Negative following full sequencing, denaturing high performance liquid chromatography or chemical cleavage.

**Table 7:** *BRCA*1 and *BRCA*2 mutation carriers

Mutation status^a^	Number of mutations	Number of families	Number of female carriers (obligate^b^)		Number of male carriers (obligate^b^)	
			All	Affected	All	Affected

*BRCA1*						
Pathogenic	62	118	282 (56)	175 (51)	115 (18)	0 (0)
Splice site	4	4	13 (3)	10 (2)	7 (0)	0 (0)
Unclassified variant	22	27	81 (4)	46 (3)	30 (2)	0 (0)
Total	88	149	376 (63)	231 (56)	152 (20)	0 (0)
*BRCA2*						
Pathogenic	58	98	237 (32)	140 (26)	85 (11)	5 (1)
Splice site	5	9	26 (2)	17 (2)	8 (5)	0 (1)
Unclassified variant	31	43	99 (2)	57 (2)	38 (3)	1 (0)
Total	94	150	362 (36)	214 (30)	131 (19)	6 (2)
Total *BRCA1 *and *BRCA2*	182	299	738 (99)	445 (86)	283 (39)	6 (2)

^a^After exclusion of common polymorphisms, mutations and variants were classified according to the kConFab classification scheme [18]; some families have pathogenic or splice site mutations, as well as an unclassified variant, some families have two unclassified variants, and some unclassified variants occur in more than one family. ^b^Obligate carriers are classified as 'definite' obligate carriers if one of their offspring and a maternal or paternal relative are both proven carriers, and 'probable' obligate carriers if they are a parent of one or more proven carriers but the other declared biological parent(s) is a proven non-carrier. Numbers of definite and probable obligate carriers (based on pedigree analysis) are in addition to tested carriers.

**Table 8:** Haplotype sharing in *BRCA1/2 *mutation negative^a ^families

Family type	*BRCA1 *haplotype shared^b^	*BRCA2 *haplotype shared^b^	*BRCA1 *and *BRCA2 *haplotype shared	Neither *BRCA1 *nor *BRCA2 *shared	Total
Female breast cancer only	13	10	10	46	79
Female breast and ovarian cancer	11	3	0	15	29
Ovarian cancer only	0	0	0	0	0
Female and male breast cancer	1	2	1	3	7
Female and male breast cancer and ovarian cancer	0	0	0	4	4
Male breast cancer only	0	0	0	0	0
Total	25	15	11	68	119

^a^Index cases screened by high-sensitivity methods. ^b^All cases from families on whom at least three blood samples were available from cases affected by breast or ovarian cancer share alleles at all markers tested.
